# The Impact of Multidimensional Warning Messages on Payment Security Behavior Across Different Scenarios

**DOI:** 10.3390/bs16030454

**Published:** 2026-03-19

**Authors:** Siyu Fan, Dongyu Liu, Te Ran, Yawen Guo, Haibo Yang

**Affiliations:** 1 Faculty of Psychology, Tianjin Normal University, Tianjin 300387, China; fansiyu@stu.tjnu.edu.cn (S.F.);; 2 Key Research Base of Humanities and Social Sciences of the Ministry of Education, Academy of Psychology and Behavior, Tianjin Normal University, Tianjin 300387, China; 3 Tianjin Key Laboratory of Student Mental Health and Intelligence Assessment, Tianjin 300387, China

**Keywords:** mobile payment, anti-fraud warning, eye tracking, security behavior

## Abstract

To ensure the security of mobile payments, anti-fraud warning messages serve as a critical defensive interface between users and potential risks. The effectiveness of their design directly influences users’ risk perceptions and security-related behaviors. The present study employed eye-tracking technology to examine the effectiveness of warning messages in mobile payment transfer scenarios and the impact of specific warning design features on user decision-making. Experiment 1 utilized a 2 (warning message: present vs. absent) × 3 (potential risk level: high, medium, low) within-subject design to test the fundamental role of warning message presence. Results indicated that the presence of warning messages significantly prolonged participants’ reaction times when selecting the transfer option, suggesting a more cautious decision-making process. Building on Experiment 1, Experiment 2 employed a 2 (warning color: red vs. blue) × 2 (warning semantic type: imperative vs. reminder) × 3 (potential risk level: high, medium, low) within-subject design and incorporated eye-tracking technology to investigate the effects of these design variables and underlying attentional mechanisms. Red warnings and imperative semantics were both found to significantly increase the likelihood of transfer rejection, with these design advantages being particularly pronounced in high-risk contexts. These findings provide empirical evidence to guide mobile payment platforms in optimizing dynamically adaptive, context-sensitive anti-fraud warning designs.

## 1. Introduction

### 1.1. The Role of Warning Messages in Mobile Payment Security and the Challenge of Habituation

The widespread adoption of mobile payments has integrated payment behaviors into daily life, but it also introduces significant information security risks, such as data theft, tampering, identity fraud, and transaction repudiation (Qiu & Yang, 2021; Yang, 2024). Within user interfaces, warning messages serve as a critical “defensive boundary,” helping users recognize threats promptly and adopt secure behaviors. Warning design is therefore essential to improving risk perception and security compliance (Ruddro & Mohna, 2023).

Warnings are notifications presented in hazardous contexts to alert users and mitigate risks (Rogers et al., 2000; Wogalter et al., 2021). Effective warnings typically feature a signal word, hazard description, consequences, and instructions, while following principles of conspicuity, legibility, comprehensibility, and motivational impact (Wogalter, 2018; Heaps & Henley, 1999).

In mobile payment scenarios, users make rapid risk assessments under time pressure to minimize transaction costs (Chang et al., 2024; Chen et al., 2020). Anti-fraud warnings act as proactive safeguards by interrupting automatic processing, prompting deliberate risk evaluation, and improving decision security (Wang et al., 2022; Buono et al., 2023).

Despite their established effectiveness in HCI research, warning habituation—reduced responsiveness to repeated warnings—frequently occurs in familiar, efficiency-oriented payment tasks (Anderson et al., 2016). Neuroimaging evidence shows attenuated neural responses after minimal exposures (Anderson et al., 2015; Kirwan et al., 2020), while longitudinal studies confirm declining compliance over time in routine contexts (Vance et al., 2018). This largely unconscious process can generalize to similar cues, causing users to ignore even critical warnings.

Consequently, before examining more complex design factors, it is essential to first establish the fundamental behavioral effect of warning presence versus absence in a controlled experimental setting.

### 1.2. Potential Risk Level and Warning Design Features

User risk decision-making is shaped by multiple interacting factors, with the magnitude of potential risk serving as a key contextual variable (Sitkin & Weingart, 1995). Loss aversion is not uniform across monetary magnitudes; in low-value contexts, sensitivity to losses may weaken or reverse (Mukherjee et al., 2017; Walasek & Stewart, 2019; Zeif & Yechiam, 2022). This raises a key question: does warning message effectiveness vary with the magnitude of potential loss? Specifically, do warnings primarily function as an “alerting” mechanism in low-risk scenarios, or as a “confirmatory” signal that reinforces caution in high-risk contexts?

Building on the established effectiveness of warnings, a practical question is how to optimize their design. Laughery (2006) stressed that effective warnings must capture attention and deliver clear, comprehensible information to support informed decisions. Hancock et al. (2020) identified two core determinants of warning effectiveness: semantic clarity (e.g., descriptive wording, explicit threat severity) and visual salience (e.g., color, placement).

The impact of warning semantic style on behavior is well documented but highly context- and user-dependent. For instance, Min (2020) found that imperative language can trigger varying emotional responses by age group, with older adults more likely to react negatively to its authoritative tone. Contextual moderation is evident: imperative warnings elicit stronger behavioral responses than informative ones in collision-avoidance tasks (Chai et al., 2022) and in fraud-interception scenarios, where direct, compelling language guides rapid action (Wang et al., 2022). In contrast, informative warnings, which explain risks, better support long-term safety awareness in educational contexts. Overall, no single style is universally superior; effectiveness is moderated by situational demands and other factors (Zhang et al., 2019; Reeder et al., 2018). Thus, warning style should align with the specific practical requirements of the context.

Color is a critical visual element in warning design. Extensive research confirms red’s effectiveness in conveying danger (Pravossoudovitch et al., 2014; Zielinska et al., 2017). ERP studies show that different background colors trigger distinct neural responses, reflecting automatic activation of associations with conventional warning colors (Yuan et al., 2021). While red and green are often contrasted, this approach has limitations: superior red performance may reflect either red’s alerting properties or green’s safety connotations reducing urgency (Bouhassoun et al., 2023; Du & Yang, 2024; Or & Wang, 2014). To isolate red’s effect, a neutral baseline color lacking strong danger or safety associations is preferable.

Eye tracking provides a precise, objective measure of attention to warnings, capturing unconscious visual processing mechanisms and subtle differences often missed by self-reports (Clay et al., 2019; Pham et al., 2018; Pastel et al., 2023). Prior work has shown dissociation between attentional metrics and behavioral intentions (Borys & Plechawska-Wójcik, 2017; Pham et al., 2018; King et al., 2021; Shi et al., 2022). In Experiment 2 of this study, eye tracking was used to record visual attention allocation during mobile payment decisions, offering direct insight into the mechanisms by which warning design features influence processing and outcomes.

### 1.3. The Present Study and Hypotheses

In summary, this study was designed to systematically investigate the effectiveness of anti-fraud warning messages in mobile payment contexts and to identify optimal design features capable of mitigating warning habituation and promoting security-related behaviors. Experiment 1 first established the fundamental effect of warning presence (vs. absence) on decision-making and examined whether this effect is moderated by the level of potential risk (high, medium, low). It was hypothesized that (H1a) the presence of a warning would result in longer reaction times, reflecting the disruption of automated processing, and that (H1b) the interaction between warning presence and risk level would be significant, with the most pronounced effects on reaction times and rejection rates observed under medium- and high-risk conditions.

Building on Experiment 1, Experiment 2 extended the investigation by examining the independent and interactive effects of warning color (red vs. blue) and semantic type (imperative vs. informative). Based on prior evidence regarding the alerting properties of red (Pravossoudovitch et al., 2014) and the persuasive advantage of imperative language in time-sensitive contexts (Chai et al., 2022), it was hypothesized that (H2a) red warnings and (H2b) imperative semantics would each increase transfer rejection rates, and (H2c) these effects would be most pronounced in high- and medium-risk scenarios. Additionally, it was predicted that (H2d) red and imperative warnings would capture greater visual attention, as indexed particularly in higher-risk contexts. Together, these steps provide empirical evidence for enhancing users’ risk perception and anti-fraud decision-making in mobile payment contexts.

## 2. Experiment 1

### 2.1. Method

#### 2.1.1. Participants

Participants were university students in China. Participants were recruited via campus posters and social media platforms (e.g., WeChat 8.0.60). Interested students contacted the researcher voluntarily, resulting in the selection of 42 university students. Their ages ranged from 17 to 30 years, with 21 male and 21 female participants. All participants had experience using smartphones and were frequent users of mobile payment applications (e.g., Alipay 10.7.30, WeChat Pay 8.0.60). To avoid potential confounding effects on risk perception, none of the participants reported prior experience with stock investment. All had normal or corrected-to-normal vision, with no color blindness or other visual impairments. Participation was voluntary, and each participant received compensation upon completion of the experiment. The distribution of demographic characteristics is presented in Table 1.

#### 2.1.2. Materials

**Figure 1:**
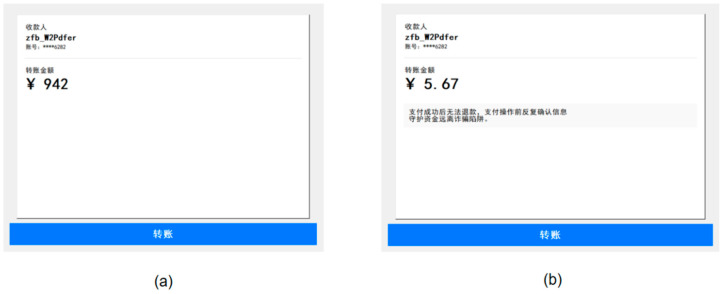
Transfer interface from Experiment 1. (**a**) transfer interfaces under high potential loss risk without a warning; (**b**) transfer interfaces under low potential loss risk with a warning. Key text translations (common to both interfaces): Top left: “收款人 zfb_W2Pdfer” → “Recipient zfb_W2Pdfer”; “账号:****6282” → “account:****6282”; “转账金额” → “Transfer amount”. Specific elements: (**b**) Warning text (right): “支付成功后无法退款，支付操作前反复确认信息，守护资金远离诈骗陷阱。” → “Once the payment is successful, it cannot be refunded. Please double-check all information before making a payment to keep your funds safe from scams.”.

To simulate the mobile payment transfer process as closely as possible, the experimental transfer interface included fields for the recipient, the transfer amount, and a payment button. To minimize the influence of the recipient on users’ risk assessment, all elements except for the warning message and the magnitude of the transfer amount remained consistent across trials (see Figure 1). To prevent habituation and automatic dismissal of the warnings, four distinct warning prompts were designed. These prompts featured slight variations in wording while maintaining semantic equivalence and similar length.

The level of potential risk was operationalized by the size of the transfer amount, categorized as large amount (500–1000), medium amount (21–499), or small amount (0–20). After the experiment, participants were asked to rate these three transfer amounts on a 5-point scale. Repeated measures ANOVA on these ratings revealed a significant main effect of amount size (*F* (2, 86) = 103.52, *p* < 0.001, η^2^ = 0.60). Post hoc comparisons using Bonferroni correction showed that ratings for small amounts (*M* = 1.29, *SD* = 0.92) were significantly lower than those for medium amounts (*M* = 2.57, *SD* = 0.76; *p* < 0.001), which in turn were significantly lower than those for large amounts (*M* = 4.00, *SD* = 1.02; *p* < 0.001). Therefore, the three transfer amounts successfully represented distinct levels of perceived potential risk, confirming the effectiveness of the experimental manipulation.

Consequently, 15 unique formal stimulus materials were prepared for each, yielding 90 formal transfer interface screens. The experiment also included 6 practice trials (using similar transfer interfaces), bringing the total number of transfer interface screens to 96. Together with one transfer success screen and one transfer failure screen, the experiment comprised a total of 98 screens.

A separate group of 42 undergraduate students was recruited to rate the experimental materials for clarity and representativeness. Clarity refers to the extent to which the stimulus material was perceived as instantly and unambiguously recognizable as depicting a “transfer” or “payment” operation, with core transactional elements (e.g., recipient, amount, action buttons) being clearly identifiable (1 = very unclear, 7 = very clear). Representativeness indicates the degree to which the stimulus material was judged to typify a genuine, realistic payment confirmation interface encountered in daily online transactions (1 = not at all representative, 7 = completely representative). The pictures showed no significant differences in either clarity (*t* (41) = 0.37, *p* > 0.050) or representativeness (*t* (41) = −0.36, *p* > 0.050).

#### 2.1.3. Apparatus

The experimental stimuli were presented on a 14.1-inch screen with a refresh rate of 60 Hz and a resolution of 1920 × 1200 pixels. A program written in Python 3.13.7 was used to control the presentation timing of the stimuli and to collect participants’ response times and key-press data.

#### 2.1.4. Experimental Design

We employed a 2 × 3 within-subject design, with warning message (present vs. absent) and potential risk level (high, medium, low) as the two factors. All participants completed every experimental condition. The decision to employ a within-subject design was primarily guided by two methodological considerations. First, to control individual differences, this design accounts for variation in risk perception and payment habits by allowing each participant to serve as their own control, thereby enhancing statistical power and reducing error variance unrelated to the experimental manipulation. Second, given the focus on how individuals adjust their decision-making processes across different warning and risk scenarios, the within-subject design enables direct comparison of within-participant behavioral changes.

#### 2.1.5. Procedure

**Figure 2:**
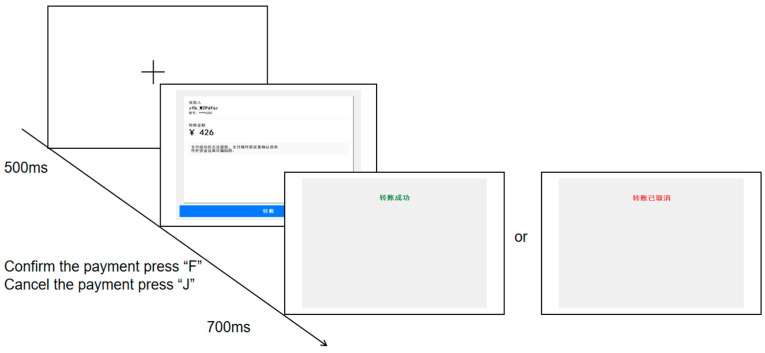
Sequence and time course of trial events for each transaction process in Experiments 1 and 2. The text in the transfer interface is the same as that in Figure 1b; please refer to Figure 1b. Feedback screens: (**Left**): “转账成功” → “Transfer successful” (green); (**Right**): “转账已取消” → “Transfer cancelled” (red).

The experiment was conducted individually in a quiet behavioral laboratory. Before the formal experiment began, the experimenter presented instructions to ensure participants understood the task. This was followed by a practice phase. Participants viewed the information on the transfer interface and made a decision based on their judgment, pressing the ‘F’ key to indicate willingness to proceed with the payment or the ‘J’ key to refuse payment, The experimental procedure is illustrated in the flowchart shown in Figure 2. Participants completed 6 practice trials to familiarize themselves with the procedure. After confirming their understanding, they proceeded with the main experiment. All trials were presented in a randomized order, and the entire session lasted approximately 8 min. Upon completion, each participant filled out a questionnaire related to the experiment.

#### 2.1.6. Data Cleaning and Statistical Analysis

Data was cleaned by excluding trials that met either of the following criteria: (1) an incorrect key-press response or (2) a reaction time that fell outside ±3 standard deviations from the mean. Repeated-measures analyses of variance (ANOVAs) and binary logistic regression analyses were subsequently conducted using SPSS (Version 26.0).

### 2.2. Results

#### 2.2.1. Payment Decisions

**Figure 3:**
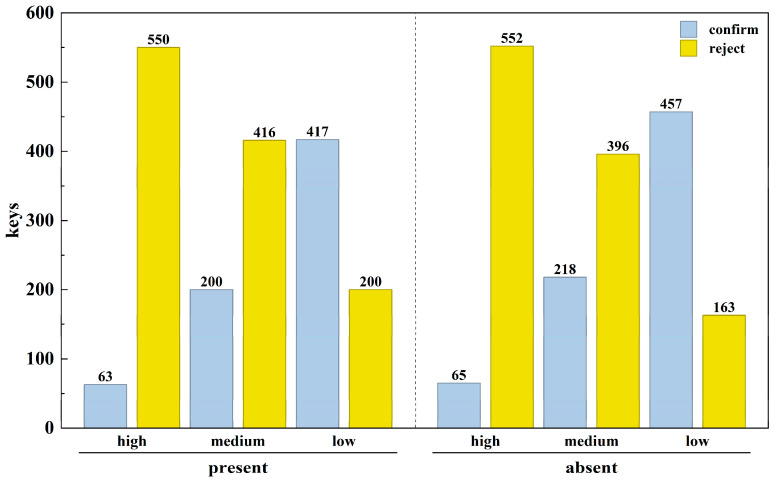
Histogram of Payment Decision Results in Experiment 1.

Binary logistic regression analysis indicated a significant main effect of potential risk level (*Wald χ*^2^ (2) = 365.196, *p* < 0.001). Relative to the high potential risk level, both the low risk level (*B* = −2.90, *SE* = 0.16, *p* < 0.001) and the medium risk level (*B* = −1.43, *SE* = 0.16, *p* < 0.001) were associated with a significantly lower probability of payment rejection. In contrast, the main effect of warning message presence was not significant (*Wald χ*^2^ = 0.022, *p* = 0.882), nor was the interaction between warning presence and potential risk level (*Wald χ*^2^(2) = 1.66, *p* = 0.436). Detailed results are presented in Figure 3 and Table 2.

#### 2.2.2. Reaction Time

The repeated-measures ANOVA revealed a significant main effect of warning message (*F* (1, 612) = 130.38, *p* < 0.001). Reaction times were significantly longer when warnings were present (*M* = 2407 ms) than when they were absent (*M* = 1662 ms). A significant main effect of potential risk level was also found (*F* (2, 1185) = 10.350, *p* < 0.001). Post hoc comparisons indicated that reaction times under medium risk (*M* = 2174 s) were significantly longer than under both high (*M* = 1909 ms) and low (*M* = 2020 ms) risk levels.

Furthermore, a significant interaction was observed between warning presence and potential risk level (*F* (2, 1185) = 7.610, *p* = 0.001). Simple-effects analysis showed that in the warning present condition, the effect of potential risk level was significant (*p* < 0.001). Here, reaction times at the medium risk level (*M* = 2671 ms) were significantly longer than at both the low (*M* = 2374 ms) and high (*M* = 2176 ms) risk levels. In contrast, under the warning absent condition, reaction times did not differ significantly across the three risk levels (range: 1640–1680 ms; *p* > 0.050). Detailed results are presented in Table 3 and Table 4.

## 3. Experiment 2

### 3.1. Method

#### 3.1.1. Participants

Forty-seven Chinese undergraduate students (21 males and 26 females) were recruited using the same sampling method and inclusion criteria as in Experiment 1. All participants received compensation for completion of the experiment. The distribution of demographic information is presented in Table 5.

#### 3.1.2. Materials

**Figure 4:**
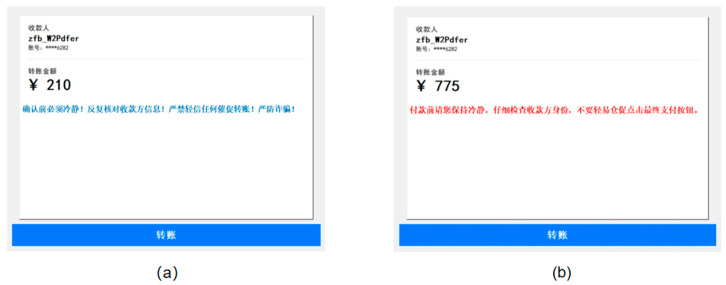
Transfer interfaces for Experiment 2. (**a**) Interface featuring an imperative warning message in blue; (**b**) Interface featuring an informative warning message in red. Key text translations (common to both interfaces): Top left: “收款人 zfb_W2Pdfer” → “Recipient zfb_W2Pdfer”; “账号: ****6282” → “account: ****6282”; “转账金额” → “Transfer amount”. Specific elements: (**a**) Warning text (blue): “确认前必须冷静！反复核对收款方信息！严禁轻信任何催促转账！严防诈骗！” → “Confirm only after calming down! Repeatedly verify the payee’s information! Do not easily trust any urge to transfer money! Be vigilant against fraud!”; (**b**) Warning text (red): “付款前请您保持冷静，仔细检查收款方身份，不要轻易仓促点击最终支付按钮。” → “Before making a payment, please stay calm, carefully check the identity of the payee, and do not rush to click the final payment button. “ Bottom button (both): “转账” → “Transfer”.

As shown in Figure 4, the experimental materials in Experiment 2 were identical to those used in Experiment 1, except for the color and semantic style of the warning messages. Based on the theoretical considerations outlined in the introduction regarding the need to avoid the confounding ‘safety’ semantics of green, blue was selected as the contrast color to red. A separate group of 35 undergraduate students was recruited to rate these materials for clarity and representativeness. No significant differences were found among the stimulus sets in either clarity (*t* (34) = 0.825, *p* > 0.050) or representativeness (*t* (34) = 0.906, *p* > 0.050).

After the experiment, participants were asked to rate the perceived directive strength of the two semantic styles on a 5-point scale. A repeated-measures ANOVA revealed a significant main effect of semantic style (*F* (1, 46) = 16.1, *p* < 0.001, η^2^ = 0.26). Ratings for the informative style (*M* = 3.4, *SD* = 0.9) were significantly lower than those for the imperative style (*M* = 4.1, *SD* = 1.01), confirming the effectiveness of the manipulation.

#### 3.1.3. Apparatus

To further investigate the implicit cognitive decision-making process, Experiment 2 employed an EyeLink 1000 Plus desktop-mounted eye tracker (SR Research Ltd., Ottawa, ON, Canada). The experimental stimuli were presented on a participant monitor with a resolution of 1920 × 1080 pixels and controlled by SR Research Experiment Builder software (version 2.5.1). The eye tracker operated at a sampling rate of 1000 Hz, ensuring high-resolution gaze tracking. Participants’ heads were stabilized using a chin rest to maintain consistency, and they were seated approximately 750 mm from the screen throughout the session. The system recorded participants’ gaze position, saccades, fixations, pupil size, and blink frequency. All participant data, including reaction times, key presses, and eye movement metrics, were automatically recorded. Subsequent analysis and extraction of data from specific screen regions were performed using Data Viewer software (Version 4.4.1). For this experiment, the area where the warning message was presented was defined as the Area of Interest (AOI).

#### 3.1.4. Experimental Design

A 2 × 2 × 3 within-subject design was used, with the factors being warning color (red vs. blue), warning semantic type (imperative vs. informative), and potential risk level (high, medium, low). Each participant completed all combinations of these conditions.

#### 3.1.5. Procedure

The experiment was conducted individually in a quiet eye-tracking laboratory. A nine-point calibration was performed at the beginning of each session. A fixation cross was displayed at the center of the screen, which participants were instructed to fixate on. Drift correction was also conducted, with a deviation of less than 1 degree considered acceptable. Subsequently, the experimenter presented the instructions to ensure participants fully understood the task, followed by a practice phase. By viewing the information on the transfer interface, participants made decisions based on their judgment, pressing the ‘F’ key to indicate willingness to proceed with the payment or the ‘J’ key to refuse payment. The experimental procedure is illustrated in Figure 2. Each trial began with the presentation of a fixation cross. Following successful calibration, the transfer interface appeared on the screen. Participants completed 10 practice trials to familiarize themselves with the procedure. Upon confirming their understanding, they proceeded to the main experiment. All trials were presented in a randomized order, with the entire session lasting approximately 20 min. After completing the experiment, each participant filled out a questionnaire.

#### 3.1.6. Data Cleaning and Statistical Analysis

The data were first cleaned by excluding trials for any of the following reasons: (1) an incorrect key-press response, (2) a value for reaction time, first fixation duration or total reading time falling beyond ±3 standard deviations from the mean. Repeated-measures analyses of variance (ANOVAs) and binary logistic regression analyses were subsequently conducted using SPSS (Version 26.0).

### 3.2. Results

#### 3.2.1. Payment Decisions

Binary logistic regression analysis revealed a significant main effect of potential risk level (*Wald χ*^2^ (2) = 433.199, *p* < 0.001). Relative to the high-risk level, both the medium-risk levels (*B* = −2.372, *SE* = 0.113, *p* < 0.001) and low-risk levels (*B* = −0.754, *SE* = 0.100, *p* < 0.001) were associated with a significantly lower probability of payment rejection.

A significant main effect of warning color was also obtained (Wald χ^2^ (1) = 107.559, *p* < 0.001). Red warnings led to a significantly higher probability of rejection compared to blue warnings (*B* = 1.171, *SE* = 0.113). Furthermore, the main effect of warning se-mantic type was significant (*Wald* χ^2^ (1) = 11.845, *p* = 0.001), with imperative warnings producing a higher probability of rejection than informative warnings (*B* = 0.356, *SE* = 0.103).

**Figure 5:**
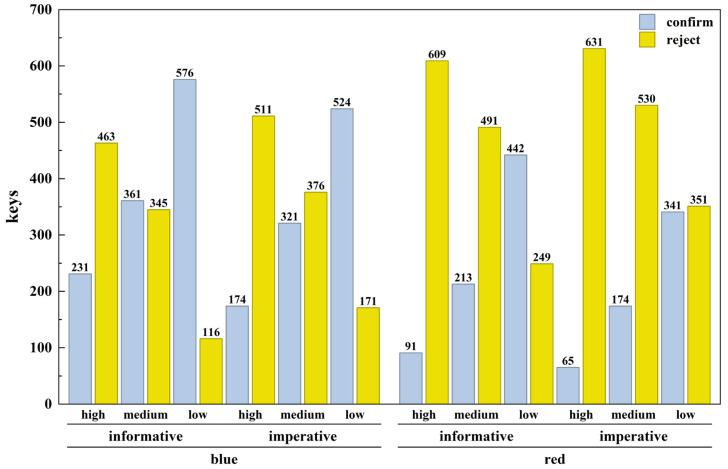
Histogram of Payment Decision Results in Experiment 2.

A significant interaction emerged between potential risk level and warning semantic type (*Wald χ*^2^ (2) = 6.803, *p* = 0.033). However, follow-up simple effects analysis indicated that the advantage of imperative over informative warnings did not reach statistical significance at either the low-risk (*p* = 0.239) or medium-risk (*p* = 0.236) levels. Simultaneously, the interaction between potential risk level and warning color approached marginal significance (*Wald χ*^2^ (2) = 5.188, *p* = 0.075). Simple-effect analysis indicated that within the medium-risk level, red warnings were associated with a higher probability of rejection than blue warnings (*B* = −0.288, *SE* = 0.113, *p* = 0.028). No other interaction effects reached statistical significance. Detailed results are presented in Figure 5 and Table 6.

#### 3.2.2. Reaction Time

A repeated-measures analysis revealed a significant main effect of potential risk level (*F* (2, 1249) = 11.763, *p* < 0.001). Post hoc comparisons indicated that reaction times at the medium risk level (*M* = 2322 ms) were significantly longer than those at both the low (*M* = 2145 ms, *p* < 0.001) and high (*M* = 2154 ms, *p* < 0.001) risk levels. No significant difference was observed between the low and high-risk levels. The main effects of warning semantic type and warning color were not significant.

A significant interaction was found between potential risk level and warning color (*F* (2, 1368) = 10.827, *p* < 0.001). Simple-effects analysis showed that for blue warnings, reaction times under low risk (*M* = 2078 ms) were significantly shorter than under both high (*M* = 2238 ms, *p* < 0.001) and medium risk (*M* = 2334 ms, *p* = 0.001), with no difference between high and medium risk levels (*p* = 0.100). In contrast, for red warnings, reaction times under high risk (*M* = 2071 ms) were significantly shorter than under both low (*M* = 2213 ms, *p* = 0.004) and medium risk (*M* = 2310 ms, *p* < 0.001), with no significant difference between low and medium risk levels (*p* = 0.100).

Furthermore, a significant interaction emerged between warning color and semantic type (*F* (1, 684) = 4.749, *p* = 0.030). For informative warnings, the difference between blue and red warnings was not significant (*p* = 0.340). However, for imperative warnings, reaction times were significantly shorter for red warnings (*M* = 2163 ms) than for blue warnings (*M* = 2233 ms, *p* = 0.040). Detailed results are presented in Table 7 and Table 8.

#### 3.2.3. Eye-Tracking Metrics

Analysis of first fixation duration revealed a significant main effect of potential risk level (*F* (2, 1326) = 3.565, *p* = 0.028). Post hoc comparisons showed that first fixations occurred significantly faster under high risk (*M* = 170 ms) than under low risk (*M* = 177 ms, *p* = 0.016) but did not differ significantly from medium risk (*M* = 175 ms, *p* = 0.100). A significant main effect of semantic type was also observed (*F* (1, 684) = 5.390, *p* = 0.021), with imperative warnings eliciting longer first fixations (*M* = 177 ms) than informative warnings (*M* = 172 ms). The main effect of warning color was not significant (*F* (1, 684) = 2.178, *p* = 0.140), and no interaction effects reached significance. Detailed results are presented in Table 9 and Table 10.

Analysis of total gaze duration showed a significant main effect of potential risk level (*F* (2, 1241) = 10.760, *p* < 0.001). Post hoc comparisons revealed that total gaze duration under the medium risk level (*M* = 1232 ms) was significantly longer than under both the low-risk (*M* = 1144 ms, *p* = 0.015) and high-risk levels (*M* = 1106 ms, *p* < 0.001). The main effects of warning semantic type and warning color were not significant. However, a significant interaction emerged between potential risk level and warning color (*F* (2, 1368) = 4.778, *p* = 0.009). Simple-effects analysis indicated that for blue warnings, total gaze duration was significantly longer under medium risk (*M* = 1226 ms) than under both low (*M* = 1110 ms, *p* = 0.001) and high risk (*M* = 1140 ms, *p* = 0.027), with no significant difference between low and high-risk levels. In contrast, for red warnings, total gaze duration was significantly shorter under high risk (*M* = 1073 ms) than under both low (*M* = 1179 ms, *p* = 0.001) and medium risk (*M* = 1237 ms, *p* < 0.001), with no significant difference between low and medium risk levels. Detailed results are presented in Table 11 and Table 12.

## 4. Discussion

### 4.1. Summary of Key Findings

Through two sequential lab experiments, this study examined the effectiveness of anti-fraud warning messages in mobile payment contexts and identified design features that mitigate habituation while promoting secure behaviors. Experiment 1 established the basic effect of warning presence versus absence. Experiment 2 used eye tracking to investigate the independent and interactive effects of warning color (red vs. blue) and semantic type (imperative vs. informative), along with underlying attentional mechanisms, while highlighting the moderating role of potential risk level. The findings emphasize the context-dependent nature of warning effectiveness and offer empirical guidance for developing dynamic, adaptive anti-fraud warning systems on mobile payment platforms.

Experiment 1 confirmed the core role of warning presence. Supporting H1, warnings significantly lengthened reaction times, disrupting automated, habitual payment processing and shifting users toward deliberate evaluation (Buono et al., 2023). This suggests well-designed warnings can retain effectiveness despite some habituation (Bravo-Lillo et al., 2013). The effect was moderated by risk level: reaction time prolongation was greatest under medium risk, weaker under low and high risk. This nonlinear pattern aligns with decision conflict literature, where intermediate attribute values or preferences produce the highest cognitive conflict and hesitation (Evans et al., 2015; Fischer et al., 2000).

Although warnings extended decision time, they did not significantly increase overall payment rejection rates overall, consistent with warning habituation in efficiency-oriented tasks (Amran et al., 2018; Vance et al., 2018). These results provide a foundation for testing specific design enhancements: while presence interrupts automatic processing, greater visual and semantic salience is required to boost compliance.

Experiment 2 built on these findings, demonstrating optimization through design features and providing objective attentional evidence via eye tracking. Supporting H2, both red warnings and imperative semantics independently increased rejection probability, with strongest effects under medium- and high-risk conditions. Red’s advantage was most pronounced at medium risk, consistent with its role as a rapid danger signal that heightens perceived urgency and threat (Pravossoudovitch et al., 2014; Yuan et al., 2021; Zielinska et al., 2017). Imperative warnings outperformed informative ones, aligning with evidence that direct, commanding language elicits stronger responses in time-sensitive fraud contexts (Chai et al., 2022; Wang et al., 2022).

Eye-tracking metrics revealed nuanced mechanisms: imperative warnings produced shorter time to first fixation, indicating faster automatic attentional capture. For total gaze duration, a color × risk interaction emerged—under high risk, red warnings elicited shorter sustained attention (efficient threat confirmation and quick rejection; Du & Yang, 2024); under medium risk, blue warnings required longer deliberative processing. These patterns extend prior eye-tracking work on warnings (Pham et al., 2018; Shi et al., 2022) and suggest a dissociation between early capture and later behavioral outcomes in mobile payments.

The red and imperative combination showed the strongest potential for accelerating secure decisions. This supports adaptive warning design that aligns with objective risk indicators (e.g., transaction amount) to optimize effectiveness across risk levels (Mukherjee et al., 2017).

### 4.2. Significance and Implications

Previous research has predominantly employed green as the control color. The present study selected blue instead to avoid the potential confound introduced by the “safety” semantics associated with green, thereby enabling a cleaner isolation of the alerting effect attributable to red as a conventional warning color. This provides more rigorous evidence for understanding the underlying mechanism of warning color efficacy (Bouhassoun et al., 2023). Furthermore, this study successfully adapted the classic framework of warning effectiveness (Rogers et al., 2000; Wogalter et al., 2021; Hancock et al., 2020) to the unique digital context of mobile payment. This addresses a gap in HCI research concerning the mechanisms of warning habituation in highly time-compressed decision tasks. The research paradigm adopted a sequential logic. Experiment 1 first established the foundational effect of warning presence versus absence, ensuring that the basic effect was not overlooked by the immediate manipulation of complex variables. Building upon this, Experiment 2 introduced multi-factor interactions and integrated eye-tracking. The systematic application of eye-tracking metrics innovatively captured early, automatic stages of warning processing (e.g., longer time to first fixation for imperative semantics and shorter total gaze duration for red under high risk). This approach moves beyond the limitations of traditional behavioral and self-report methods by revealing the dissociation between subjective evaluation and objective attention (Shi et al., 2022), thereby addressing a common shortcoming in prior studies that often focused on single variables or subjective reports.

This study offers concrete, actionable recommendations for optimizing anti-fraud interface design in mobile payment platforms. In the context of widespread mobile payment adoption and the high prevalence of telecom fraud (Chang et al., 2024; Chen et al., 2020), platforms should prioritize the use of red warnings paired with imperative semantics to maximize attentional capture and compliance with safe behavior. Furthermore, the development of risk-adaptive warning systems—which dynamically adjust salience and content based on transaction context—can significantly reduce users’ susceptibility to fraud and mitigate financial losses for platforms. These findings can also be extended to broader digital-risk scenarios, such as browser security warnings and phishing email interception, thereby contributing to the enhancement of public risk awareness in increasingly digitalized environments.

### 4.3. Limitations and Future Research

The study was conducted in a laboratory setting using static screenshots of transfer interfaces, rather than simulating the operational workflow of an actual mobile payment application. As a result, the motivational involvement of participants and the real financial consequences were reduced compared to genuine transactional contexts. Future research should shift toward testing within real application environments or employ high-fidelity simulations that incorporate real monetary incentives or loss penalties to improve ecological validity. Second, the participant sample was limited to undergraduate students from university, which restricted the age range represented. The risk perception and mobile payment habits of this group may differ considerably from those of other demographics, such as middle-aged or older adults or professional investors. Subsequent studies should therefore expand recruitment to broader population segments, with particular attention to groups that are vulnerable to telecom fraud. However, despite the implementation of procedural controls such as trial randomization, within-subject designs remain susceptible to potential confounding factors, including demand characteristics and fatigue effects. Future research could adopt a mixed experimental design to validate the robustness of the present findings. Furthermore, future work could integrate physiological and neuroscientific measures—such as event-related potentials (ERPs), functional magnetic resonance imaging (fMRI), or heart rate monitoring—to obtain deeper insight into the neural mechanisms underlying warning processing. Such approaches would support a more nuanced understanding of how warnings are processed and how their effectiveness can be enhanced, ultimately enabling more precise and personalized warning design.

## 5. Conclusions

In mobile payment contexts, effective anti-fraud warning design transcends simple information delivery and requires a holistic integration of visual and semantic features. Specifically, well-designed warnings can disrupt users’ automated processing and promote more deliberate, cautious decision-making. Compared to blue warnings, red warnings lead to higher security behavior compliance; similarly, imperative semantics outperform informative semantics in guiding users toward safe actions. The effectiveness of warning color is moderated by risk level, with red particularly enhancing users’ perception of and response to high-risk situations. Eye-tracking measures further provide objective evidence of the underlying attentional mechanisms. Together, these findings offer empirical support and practical design guidance for mobile payment platforms to build more efficient and precise risk–intervention interfaces.

## Figures and Tables

**Table 1:** The distribution of demographic information of subjects in Experiment 1.

Demographic Information	Information of All Subjects
Number	42
Gender	Male: 21 (50%) Female: 21 (50%)
Age	19.27 (±2.368)
Native Language	Mandarin Chinese
Education Level	In college or graduate school

**Table 2:** Binary Logistic Regression Analysis for Payment Decisions in Experiment 1.

Predictor	*B*	*SE*	*Wald’s χ^2^*	*df*	*p*	*OR*	*95% CI for OR*
Constant	2.167	0.133	265.383	1	<0.001	8.730	
Alert (ref = absent)							
Alert (present)	−0.028	0.187	0.022	1	0.882	0.973	[0.675, 1.403]
Risk level (ref = high risk)			365.196	2	<0.001		
Low risk	−2.902	0.158	335.562	1	<0.001	0.054	[0.040, 0.075]
Medium risk	−1.434	0.158	81.990	1	<0.001	0.238	[0.175, 0.325]
Alert × Risk level			1.661	2	0.436		
Low risk × Alert	−0.269	0.225	1.425	1	0.233	0.764	[0.492, 1.188]
Medium risk × Alert	−0.108	0.222	0.235	1	0.628	0.90	[0.581, 1.388]

**Table 3:** Descriptive statistics for Reaction Time in Experiment 1.

Risk Level	Present	Absent
High	2176 ± 2124	1641 ± 1449
Medium	2670 ± 2726	1678 ± 1324
Low	2374 ± 2141	1665 ± 1386

**Table 4:** Repeated-Measures ANOVA for Reaction Time in Experiment 1.

Source	*Type III Sum of Squares*	*df*	*MS*	*F*	*p*	*Partial η^2^*
Alert	510.916	1	510.916	130.383	<0.001	0.176
Risk level	43.658	2	21.829	10.350	<0.001	0.017
Alert × Risk level	32.732	2	16.366	7.610	0.001	0.012

**Table 5:** The distribution of demographic information of subjects in Experiment 2.

Demographic Information	Information of All Subjects
Number	47
Gender	Male: 21 (44.7%) Female: 26 (55.3%)
Age	20.26 (±1.939)
Native Language	Mandarin Chinese
Education Level	In college or graduate school

**Table 6:** Binary Logistic Regression Analysis for Payment Decisions in Experiment 2.

Predictor	*B*	*SE*	*Wald’s χ^2^*	*df*	*p*	*OR*	*95% CI for OR*
Constant	0.707	0.076	86.502	1	<0.001	2.028	
Risk level (ref = high risk)			433.199	1	<0.001		
Low risk	−2.327	0.113	422.606	1	<0.001	0.098	[0.078, 0.122]
Medium risk	−0.754	0.100	56.635	1	<0.001	0.471	[0.387, 0.573]
Informative	0.356	0.103	11.845	1	0.001	1.428	[1.166, 1.749]
Blue	1.171	0.113	107.559	1	<0.001	3.225	[2.584, 4.023]
Risk level × Tone			6.803	2	0.033		
Low risk × Informative	0.157	0.133	1.386	1	0.239	1.169	[0.901, 1.518]
Medium risk × Informative	−0.150	0.127	1.403	1	0.236	0.860	[0.671, 1.104]
Risk level × Color			5.188	2	0.075		
Low risk × Blue	−0.115	0.136	0.714	1	0.398	0.892	[0.683, 1.163]
Medium risk × Blue	−0.288	0.131	4.807	1	0.028	0.750	[0.580, 0.970]
Blue × Informative	0.070	0.103	0.468	1	0.494	1.073	

**Table 7:** Descriptive statistics for Reaction Time in Experiment 2.

Risk Level	Color	Informative	Imperative
High	Blue	2244 ± 1556	2234 ± 1591
	Red	2092 ± 1401	2050 ± 1431
Medium	Blue	2308 ± 1491	2361 ± 1520
	Red	2321 ± 1511	2300 ± 1491
Low	Blue	2039 ± 1239	2118 ± 1382
	Red	2286 ± 1522	2140 ± 1379

**Table 8:** Repeated-Measures ANOVA for Reaction Time in Experiment 2.

Source	*Type III Sum of Squares*	*df*	*MS*	*F*	*p*	*Partial η^2^*
Risk level	54,240,960.622	2	27,120,480.311	11.763	<0.001	0.017
Color	763,643.914	1	763,643.914	0.517	0.472	0.001
Tone	438,447.309	1	438,447.309	0.340	0.560	<0.001
Risk level × Color	31,260,421.042	2	15,630,210.521	10.827	<0.001	0.016
Risk level × Tone	966,148.387	2	483,074.194	0.315	0.730	<0.001
Color × Tone	6,230,964.207	1	6,230,964.207	4.749	0.030	0.007
Risk level × Color × Tone	3,490,879.951	2	1,745,439.975	1.350	0.259	0.002

**Table 9:** Descriptive statistics for First Fixation Duration in Experiment 2.

Risk Level	Color	Informative	Imperative
High	Blue	171 ± 80	172 ± 75
	Red	168 ± 77	172 ± 83
Medium	Blue	172 ± 73	174 ± 105
	Red	170 ± 78	186 ± 139
Low	Blue	173 ± 75	175 ± 80
	Red	178 ± 133	181 ± 100

**Table 10:** Repeated-Measures ANOVA for First Fixation Duration in Experiment 2.

Source	*Type III Sum of Squares*	*df*	*MS*	*F*	*p*	*Partial η^2^*
Risk level	61,696.333	2	30,848.167	3.565	0.030	0.005
Color	18,112.993	1	18,112.993	2.178	0.140	0.003
Tone	44,734.669	1	44,734.669	5.390	0.021	0.008
Risk level × Color	19,564.355	2	9782.177	1.111	0.329	0.002
Risk level × Tone	19,289.144	2	9644.572	1.164	0.312	0.002
Color × Tone	21,831.243	1	21,831.243	2.406	0.121	0.004
Risk level × Color × Tone	21,842.562	2	10,921.281	1.299	.273	0.002

**Table 11:** Descriptive statistics for Total Gaze Duration in Experiment 2.

Risk Level	Color	Informative	Imperative
High	Blue	1159 ± 1033	1121 ± 941
	Red	1072 ± 866	1074 ± 1000
Medium	Blue	1203 ± 992	1250 ± 1031
	Red	1238 ± 1025	1237 ± 1016
Low	Blue	1096 ± 856	1126 ± 638
	Red	1237 ± 1075	1121 ± 513

**Table 12:** Repeated-Measures ANOVA for Total Gaze Duration in Experiment 2.

Source	*Type III Sum of Squares*	*df*	*MS*	*F*	*p*	*Partial η^2^*
Risk level	22,645,027.822	2	11,322,513.911	10.760	<0.001	0.015
Color	33,855.125	1	33,855.125	0.053	0.819	<0.001
Tone	336,256.141	1	336,256.141	0.543	0.462	0.001
Risk level × Color	6,343,337.898	2	3,171,668.949	4.778	0.009	0.007
Risk level × Tone	1,510,368.692	2	755,184.346	1.197	0.302	0.002
Color × Tone	1,365,672.300	1	1,365,672.300	2.370	0.124	0.003
Risk level × Color × Tone	3,014,587.069	2	1,507,293.534	2.648	0.071	0.004

## Data Availability

The data presented in this study are available upon request from the corresponding author. The data is not publicly available due to participant confidentiality and institutional ethical guidelines.
